# Storage-Induced Micro-Erythrocytes Can Be Quantified and Sorted by Flow Cytometry

**DOI:** 10.3389/fphys.2022.838138

**Published:** 2022-02-23

**Authors:** Mickaël Marin, Sandy Peltier, Youcef Hadjou, Sonia Georgeault, Michaël Dussiot, Camille Roussel, Olivier Hermine, Philippe Roingeard, Pierre A. Buffet, Pascal Amireault

**Affiliations:** ^1^INSERM, BIGR, Université de Paris and Université des Antilles, Paris, France; ^2^Institut National de la Transfusion Sanguine, Paris, France; ^3^Laboratoire d’Excellence GR-Ex, Paris, France; ^4^Plateforme des Microscopies, Infrastructures de Recherche en Biologie Santé et Agronomie, Programme Pluriformation Analyse des Systèmes Biologiques, Tours, France; ^5^U1163, Laboratory of Cellular and Molecular Mechanisms of Hematological Disorders and Therapeutic Implications, INSERM, Université de Paris, Paris, France; ^6^AP-HP, Laboratoire d’Hématologie, Hôpital Necker-Enfants Malades, Paris, France; ^7^Département d’Hématologie, Hôpital Necker-Enfants Malades, Assistance Publique–Hôpitaux de Paris, Paris, France; ^8^U1259, Centre Hospitalier Régional Universitaire de Tours, Morphogenèse et Antigénicité du VIH et des Virus des Hépatites, INSERM, Université de Tours, Tours, France; ^9^AP-HP, Paris, France

**Keywords:** red blood cell (RBC), RBC storage, RBC morphology, flow cytometry, imaging flow cytometry (IFC), RBC storage lesion

## Abstract

Refrigerated storage of red cell concentrates before transfusion is associated with progressive alterations of red blood cells (RBC). Small RBC (type III echinocytes, sphero-echinocytes, and spherocytes) defined as storage-induced micro-erythrocytes (SME) appear during pretransfusion storage. SME accumulate with variable intensity from donor to donor, are cleared rapidly after transfusion, and their proportion correlates with transfusion recovery. They can be rapidly and objectively quantified using imaging flow cytometry (IFC). Quantifying SME using flow cytometry would further facilitate a physiologically relevant quality control of red cell concentrates. RBC stored in blood bank conditions were stained with a carboxyfluorescein succinimidyl ester (CFSE) dye and incubated at 37°C. CFSE intensity was assessed by flow cytometry and RBC morphology evaluated by IFC. We observed the accumulation of a CFSE*^high^* RBC subpopulation by flow cytometry that accounted for 3.3 and 47.2% at day 3 and 42 of storage, respectively. IFC brightfield images showed that this CFSE*^high^* subpopulation mostly contains SME while the CFSE*^low^* subpopulation mostly contains type I and II echinocytes and discocytes. Similar numbers of SME were quantified by IFC (based on projected surface area) and by flow cytometry (based on CFSE intensity). IFC and scanning electron microscopy showed that ≥95% pure subpopulations of CFSE*^high^* and CFSE*^low^* RBC were obtained by flow cytometry-based sorting. SME can now be quantified using a common fluorescent dye and a standard flow cytometer. The staining protocol enables specific sorting of SME, a useful tool to further characterize this RBC subpopulation targeted for premature clearance after transfusion.

## Introduction

During hypothermic pre-transfusion storage, part of the RBC metabolism shifts from glycolysis to the pentose phosphate pathway after 10–14 days of storage, leading to a progressive decrease of the intracellular ATP level (D’Alessandro et al., 2015; Bordbar et al., 2016). The fragilized RBC is thus less capable of coping with the oxidative stress generated by storage, further affecting RBC integrity (Reisz et al., 2016). These metabolic and oxidative stresses contribute to the progressive modifications of RBC properties (Yoshida et al., 2019). Among those, RBC morphology is a well-documented and key RBC property altered during storage.

A number of techniques are used to explore RBC morphology. The pioneer work of Bessis (1972, 1974) using scanning electron microscopy defined RBC morphological categories. This technique is still used to assess RBC morphology during storage (Berezina et al., 2002; Zehnder et al., 2008; Antonelou et al., 2012; Blasi et al., 2012; D’Alessandro et al., 2012; Roussel et al., 2021).

Digital holographic microscopy was recently used to explore RBC morphology along storage (Bardyn et al., 2017). Light microscopy can also be used to observe fixed (Högman et al., 1985; Tchir et al., 2013; Reinhart et al., 2015) or unfixed cells in a physiological medium (Roussel et al., 2017; Lu and Shevkoplyas, 2020). RBC morphology is indeed sensitive to RBC intrinsic properties and to the suspension medium (Bessis, 1972). Imaging flow cytometry (IFC) rapidly acquires tens of thousands of images of unfixed cells in a physiological medium and identifies a well-demarcated subpopulation of morphologically altered and smaller RBC, comprising type III echinocytes, spheroechinocytes, and spherocytes (Roussel et al., 2017). These small RBC, defined as storage-induced micro-erythrocytes (SME), accumulate during storage, reaching a mean proportion of 24% of the entire RBC population at day 42 and their proportion negatively correlates with post-transfusion recovery in healthy volunteers (Roussel et al., 2021). Furthermore, the capacity of IFC to discriminate fluorescently stained RBC allowed to observe their clearance from circulation upon *ex vivo* perfusion in human spleens and *in vivo* transfusion in mice (Roussel et al., 2021). The proportion of SME is thus a relevant storage quality marker that identifies the erythrocyte subpopulation targeted for rapid post-transfusion clearance. This study aimed to develop an alternative method of SME quantification and sorting using standard flow cytometry. Obtaining pure subpopulations of SME or morphologically normal erythrocytes would indeed greatly facilitate the exploration of the intrinsic alterations of RBC inducing their clearance.

## Materials and Methods

### Red Blood Cells Concentrate Collection and Storage

Eight leukoreduced RBC concentrates provided by the Etablissement Français du Sang (French blood banking system) from healthy donors were stored in saline-adenine-glucose-mannitol (SAGM) solution at 2–6°C for 42 days. Samples were aseptically collected and analyzed at defined time-points (days 3, 21, 28, 35, and 42).

### Carboxyfluorescein Diacetate Succinimidyl Ester Staining

Red blood cells were washed once in phosphate buffer saline (PBS) and stained with CFDA-SE (5.5 millions RBC/mL, 0.05 μM CFSE in PBS) for 20 min at 37°C. Non-fluorescent CFDA-SE diffuses passively into cells and is rapidly processed by cellular esterases resulting in high fluorescent carboxyfluorescein succinimidyl esters (CFSE) that bind cellular components. RBC were then centrifuged, washed once in RPMIc (RPMI 1640 supplemented with 10% FBS, 1% Antibiotic/Antimycotic solution) to remove excess of CFDA-SE and incubated overnight in RPMIc at 22 millions RBC/mL. Following incubation, RBC were centrifuged, resuspended in a fresh RPMIc solution and stored at 4°C until analysis.

### Flow Cytometry Analysis

Carboxyfluorescein succinimidyl ester stained RBC were analyzed using FACSCanto II (BD Biosciences) by recording 50,000 events. First, single cells were selected using morphological parameters (FSC-H *vs.* FSC-A). CFSE*^low^* and CFSE*^high^* subpopulations were gated according to their size (FSC-W) and their CFSE intensity (collected in FITC channel).

### Imaging Flow Cytometry Analysis

Imaging flow cytometry was performed by ImageStream X Mark II (Amnis^®^ Flow Cytometry, Luminex, Seattle, WA, United States) to determine RBC dimensions and morphology as described (Roussel et al., 2017). RBC were suspended at 1% hematocrit (Hct) just before acquisition in a Krebs-albumin solution (Krebs-Henseleit buffer, Sigma-Aldrich) modified with 2 g of glucose, 2.1 g of sodium bicarbonate, 0.175 g of calcium chloride dehydrate, and 5 g of lipid-rich bovine serum albumin (AlbuMAX II, Thermo Fisher Scientific) for 1 L of sterile water (pH 7.4). Images (x60 magnification) were recorded (INSPIRE software, AMNIS) by the brightfield and FITC channels to be then processed by a dedicated computer software [IDEAS (version 6.2); Amnis]. Focused cells and single cells were, respectively, selected using the features gradient RMS_M01_Ch01 and Aspect ratio_M01_Ch01 versus Area_M01_Ch01. Front views were selected using the feature Circularity_Object (M01, Ch01, Tight) and projected surface area was determined using the feature Area_Object (M01, Ch01, Tight). At least 6,000 front views of focused single RBC/condition were analyzed. SME proportion and CFSE intensity were determined independently for each donor, using the nadir of the bimodal frequency histograms as the gating boundary.

### Cell Sorting

Sorting of CFSE*^low^* and CFSE*^high^* cells was performed using MA900 Cell Sorter (Sony) with a 100 μm sorting-chip at the maximum speed of 10,000 events per second in semi-purity mode. Unsorted RBC were selected using BSC-A *vs.* FSC-A then CFSE*^low^* and CFSE*^high^* subpopulations were gated according to their size (FSC-W) and their CFSE intensity (collected in FITC channel). Cell doublets were excluded by using FSC-H *vs.* FSC-A and target cells were collected in tubes containing 1 mL of RPMIc, then centrifuged and resuspended in RPMIc to be stored at 4°C until analysis.

### Scanning Electron Microscopy

Samples were fixed by incubation (minimum 24 h) in a mix of 4% paraformaldehyde and 1% glutaraldehyde diluted in 0.1 M of phosphate buffer (pH, 7.3) at 4°C, then washed in phosphate buffer, and postfixed by 1-h incubation with 2% osmium tetroxide. Samples were then fully dehydrated in a graded series of ethanol solutions and dried by hexamethyldisilazane. Finally, samples were coated with 4 nm of carbon using a GATAN PECS 682 apparatus before observation under a Zeiss Ultra plus field emission-scanning electron microscope (ZEISS).

### Statistical Analysis

Data were analyzed using GraphPad Prism version 9.2.0 for Windows (GraphPad Software, San Diego, CA, United States). To compare two groups of subpopulations (CFSE*^low^ vs.* CFSE*^high^* intensities or SME *vs.* normal erythrocytes), Wilcoxon tests, for non-parametric and paired data, were applied. To compare means of morphologically altered RBC over time, two-way ANOVA with the Geisser–Greenhouse correction was performed with Sidak’s multiple comparison test. Simple linear regression and a correlation test of Spearman were used to assess correlation between the two quantification techniques of morphologically altered RBC. A *P*-value < 0.05 was considered statistically significant.

## Results

### Incubation-Related Bimodality in Carboxyfluorescein Diacetate Succinimidyl Ester Staining Intensity of Red Blood Cells Stored in Blood Bank Conditions

**FIGURE 1:**
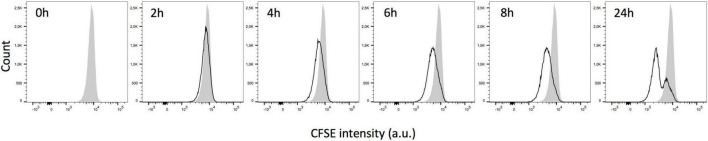
Bimodality in carboxyfluorescein diacetate succinimidyl ester (CFDA-SE) staining intensity of RBC stored in blood bank conditions. Representative frequency plots of CFSE fluorescence intensity for RBC stored for 42 days in SAGM either immediately after CFDA-SE staining (gray histogram) and during the 24 first hours of incubation at 37°C (black line).

Red blood cells, sampled from long-stored RBC concentrates, were stained using CFDA-SE and then incubated at 37°C in RPMIc medium. Immediately after CFDA-SE staining, CFSE fluorescence intensity exhibited a unimodal distribution (0 h, gray histogram, Figure 1). Incubation of the stained RBC at 37°C led to a progressive decline of fluorescence intensity until 8 h of incubation (black curves, Figure 1). Following an overnight incubation, the CFSE intensity showed a clear bimodality separating two RBC subpopulations (Panel 24 h, Figure 1). The bimodality in CFSE staining was not visible when fresh RBC were observed or when RBC were incubated at 4°C after staining (data not shown). These results show that the CFDA-SE staining protocol with an overnight incubation identifies a discrete RBC subpopulation present in long-stored RBC.

### CFSE*^high^* Erythrocytes Correspond to a Morphologically Altered Subpopulation That Accumulates During Pretransfusion Storage

**FIGURE 2:**
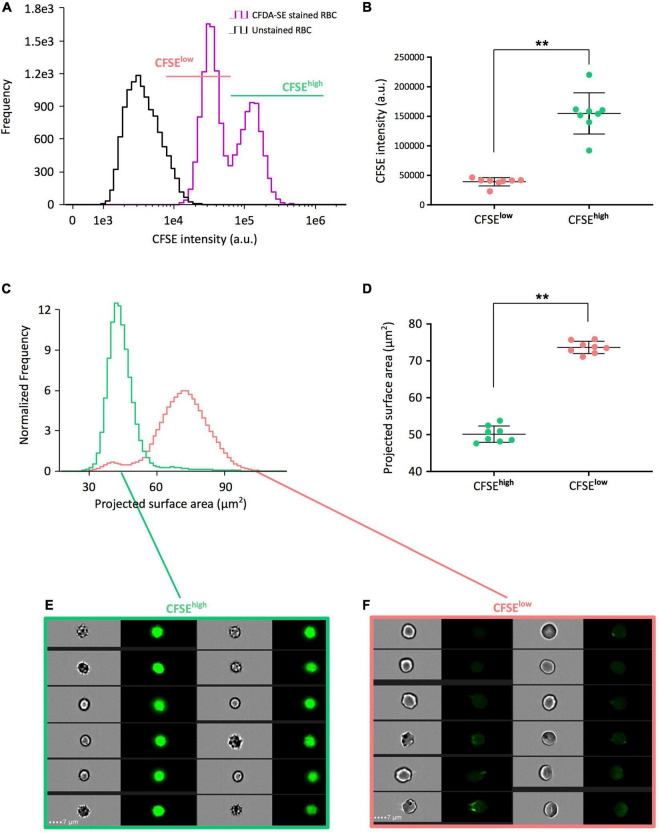
CFSE*^high^* RBC correspond to a morphologically altered subpopulation that accumulates during pretransfusion storage. **(A)** Representative CFSE intensity frequency plots of unstained (black line) and CFDA-SE stained (purple line) long-stored RBC allowing to select CFSE*^low^* (pink) and CFSE*^high^* (light green) subpopulations by the nadir point of the bimodality of frequency plots using imaging flow cytometry (IFC). **(B)** Comparison of the mean CFSE fluorescence intensities of each subpopulation obtained from eight red cell concentrates (RCC) stored 42 days and determined by IFC. **(C)** Representative projected surface area frequency plots of the CFSE*^low^* and CFSE*^high^* subpopulations previously selected in **(A)**. **(D)** Comparison of the mean projected surface area from the CFSE subpopulations previously selected in **(B)** from the eight long-stored RCC. Representative brightfield and fluorescence images from CFSE*^high^* **(E)** showing mostly echinocytes III, spheroechinocytes and spherocytes, and CFSE*^low^* subpopulations **(F)** showing discocytes, echinocytes I and echinocytes II. Scale bars represent 7 μm. Results are presented as mean ± SD in **(B,D)** and tests of Wilcoxon for non-parametric and paired data were applied for group comparisons (**p = 0.0078).

We next used imaging flow cytometry, that enables simultaneous analysis of fluorescence and morphological parameters, on eight RBC concentrates stored for 42 days. A bimodal distribution of CFDA-SE staining was detected (purple curve, Figure 2A) and visible in 8/8 RBC concentrates. Segregating CFSE*^low^* RBC (pink line, Figure 2A) from CFSE*^high^* RBC (light green line, Figure 2A) was performed independently for each donor, using the nadir of the bimodal frequency histograms as the gating boundary. The mean fluorescence intensity of CFSE*^high^* RBC (154729 a.u. ± 34931) was statistically higher than that of the CFSE*^low^* subpopulation (39055 a.u. ± 6980; *p* = 0.0078; Figure 2B).

We next determined the projected surface area of each subpopulation along storage to discriminate morphologically altered RBC, named storage-induced micro-erythrocytes (SME), from morphologically normal erythrocytes (Roussel et al., 2017, 2021). Comparison of the distribution of projected surface area between the two subpopulations showed that CFSE*^high^* RBC have a lower projected surface area than RBC from the CFSE*^low^* subpopulation (Figure 2C). CFSE*^high^* RBC were significantly smaller than CFSE*^low^* RBC (projected surface area of 50.1 μm^2^ ± 2.2 versus 73.7 μm^2^ ± 1.7; *p* = 0.0078; Figure 2D). Morphologic analysis of IFC brightfield images showed that the CFSE*^high^* RBC were predominantly echinocytes III, spheroechinocytes and spherocytes (Figure 2E) while the CFSE*^low^* subpopulation was composed mostly of discocytes and echinocytes I and II (Figure 2F). Reciprocally, selection of SME and morphologically normal erythrocytes (by their projected surface area) confirmed that most SME are CFSE*^high^* and that morphologically normal erythrocytes are CFSE*^low^* (Supplementary Figure 1). The gating strategy to segregate CFSE*^low^* and CFSE*^high^* subpopulations could be improved using a size parameter, in addition to CFSE intensity (Supplementary Figure 2).

### Carboxyfluorescein Diacetate Succinimidyl Ester Staining Enables the Flow Cytometry-Based Quantification of Storage-Induced Micro-Erythrocytes Along Storage

**FIGURE 3:**
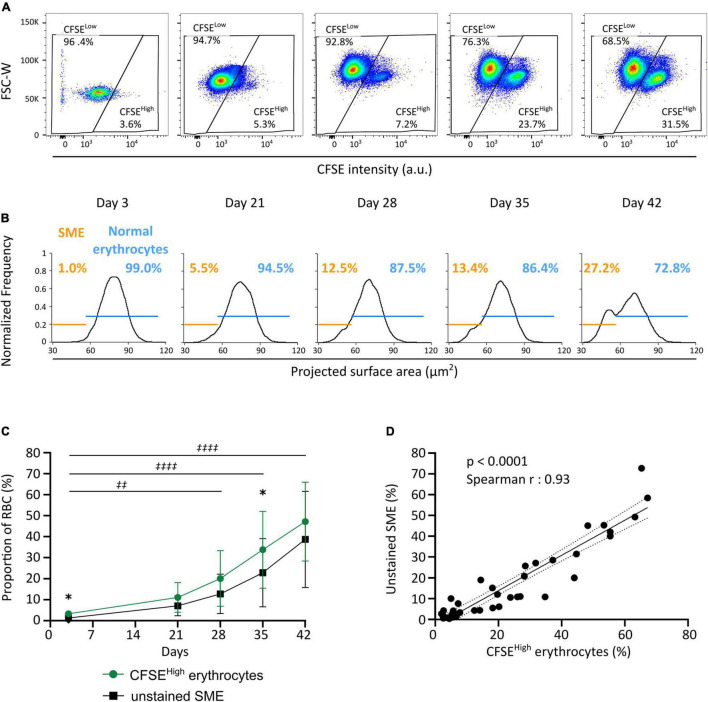
CFDA-SE staining allows the quantification of SME by flow cytometry. **(A)** Representative density plots allowing the quantification of CFSE*^low^* and CFSE*^high^* subpopulations along storage by flow cytometry. **(B)** Representative projected surface area frequency plots for unstained RBC allowing quantification of morphologically normal erythrocytes (blue) and SME (orange) along storage using imaging flow cytometry (IFC). **(C)** Evolution of the proportion of CFSE*^high^* (green line) and unstained SME subpopulations (black line) during red cell concentrates storage (*n* = 8). **(D)** Correlation between results obtained across storage with either of the two quantification techniques: i.e., CFSE*^high^* erythrocytes and SME that accumulate along storage [Spearman *r* = 0.93, *p* < 0.0001; best-fit line is presented (black solid line) with its 95% confidence interval (black dotted lines)]. Results in **(C)** are represented as mean ± SD (vertical bars) and a two-way ANOVA, with the Geisser–Greenhouse correction followed by a Sidak’s multiple comparison, compared both techniques at each time point (*p < 0.05) or the accumulation of CFSE*^high^* subpopulation along storage *vs.* day 3 (‡‡*p* = 0.0011; ‡‡‡‡*p* < 0.0001).

We next evaluated the evolution of RBC morphology during storage by flow cytometry using the CFDA-SE staining protocol and compared these observations to the proportion of SME detected by IFC on unstained RBC (Roussel et al., 2017, 2021). A CFSE*^high^* subpopulation (now gated using FSC-W and CFSE intensity) accumulated along storage (Figure 3A) in the eight RBC concentrates studied (green line, Figure 3C). A SME subpopulation (using the nadir of the projected surface area bimodality) also accumulated along storage (Figure 3B) in the eight RBC concentrates studied (black line, Figure 3C). For all donors, the CFSE*^high^* subpopulation accumulated upon storage from 3.3 ± 1.4% on day 3 to 47.2 ± 18.8% on day 42 (green line, Figure 3C), with marked inter-donor variability. Similarly, the SME subpopulation accumulated upon storage from 1.3 ± 0.8% on day 3 to 38.7 ± 22.9% on day 42 (black line, Figure 3C). The proportion of morphologically altered RBC followed a similar evolution along storage with both techniques, increasing more rapidly after day 21. The proportion of CFSE*^high^* RBC was slightly higher than the proportion of SME at each time point, reaching statistical significance on day 3 and 35 of storage. There was a very strong correlation between the proportion of CFSE*^high^* RBC determined using flow cytometry and the proportion of SME determined by IFC (*p* < 0.0001; Spearman *r* = 0.93; *r*^2^ = 0.88; Figure 3D).

### Pure Subpopulations of Morphologically Normal Erythrocytes or Storage-Induced Micro-Erythrocytes Can Be Obtained by Flow Cytometry-Based Sorting

**FIGURE 4:**
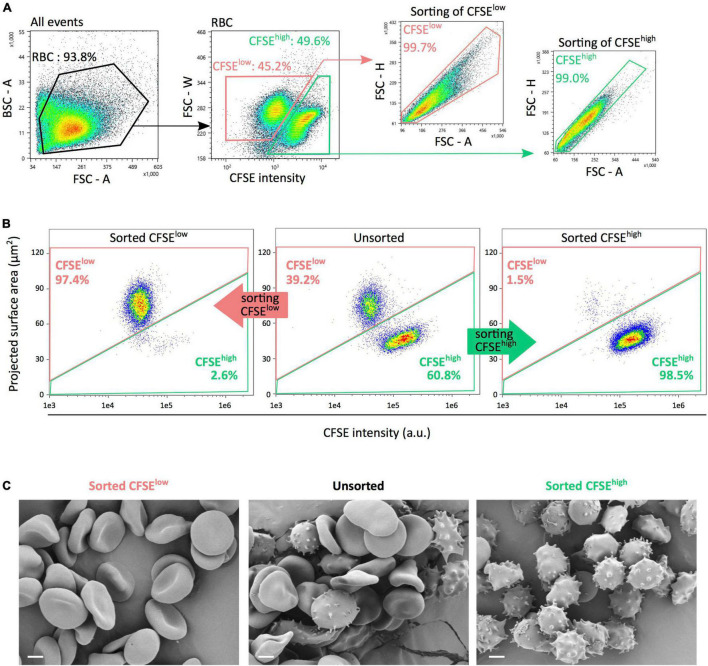
FACS-based sorting generates pure subpopulations of either morphologically normal erythrocytes or SME. **(A)** Flow cytometry sorting gating strategy for sorting, using fluorescence intensity and morphological parameters to select CFSE*^low^* (pink) and CFSE*^high^* (light green) RBC subpopulations. Morphological criteria comprised the forward scatter (FSC) or back scatter (BSC) signals and their respective parameter of area (A), height (H), and width (W). **(B)** Representative imaging flow cytometry density plots of unsorted (middle panel), sorted CFSE*^low^* (left panel), and CFSE*^high^* (right panel) subpopulations illustrating each preparation purity. **(C)** Scanning electron microscopy images showing typical morphological patterns (discocytes, echinocytes and spheroechinocytes, or balanced distribution) of RBC in each unsorted or sorted subpopulations. CFSE*^low^* and CFSE*^high^* subpopulations are presented in pink and light green, respectively. Scale bars represent 2 μm.

A flow cytometry gating strategy was next used to sort CFSE*^low^* and CFSE*^high^* RBC subpopulations (Figure 4A). IFC was used to evaluate the content of each preparation, using fluorescence intensity and projected surface area. In Figure 4B, representative density plots of unsorted (middle panel), sorted CFSE*^low^* (left panel), and CFSE*^high^* (right panel) subpopulations illustrate the average purity obtained. From five separate experiments conducted on RBC stored 42 days, preparations of CFSE*^low^* and CFSE*^high^* contained, respectively, a mean proportion of RBC of interest of 96.4 ± 1.4% and 97.9 ± 2.0%. Scanning electron microscopy images confirmed that a majority of morphologically normal erythrocytes (as discocytes and echinocytes I) were found in CFSE*^low^* subpopulations (Figure 4C, left panel) while a majority of SME (echinocytes III, spheroechinocytes and spherocytes) were present in CFSE*^high^* subpopulation (right panel) with a mix of these morphologies in the unsorted fraction (middle panel).

## Discussion

We describe here a simple staining flow cytometry-based protocol to quantify SME that accumulate during pretransfusion storage. In addition, pure subpopulations of SME and long-stored morphologically normal erythrocytes can now be obtained using flow cytometry-based sorting.

We observed that CFDA-SE stained long-stored RBC show a bimodality in fluorescence intensity after an incubation at 37°C. This bimodality was not observed immediately after staining nor after a 24 h incubation at 4°C suggesting that it could be due to an enzymatic process. Non-fluorescent CFDA-SE diffuses passively into cells and is rapidly processed by cellular esterases resulting in high fluorescent CFSE. The resulting molecule is less permeant to cell membrane and highly reactive to free cellular amines, which leads to its spontaneous covalent binding to cellular components, mostly intracellular proteins (Banks et al., 2013). Thus, the persistence of CFSE labeling in cells is expected to depend predominantly on protein turn-over and its natural fluorescence decay, making it a useful and widely used cell division tracker (Lyons et al., 2013).

In our study, the decrease in CFSE fluorescence intensity was not evenly distributed across all long-stored RBC, revealing instead two well-demarcated RBC subpopulations. IFC images showed that RBC with stable and thus higher CFSE fluorescence display marked morphological alterations comprising mostly SME, while RBC with decreased CFSE fluorescence are morphologically normal. This suggests that an enzymatic and/or metabolic process is lost in SME and preserved in morphologically normal erythrocytes. Even though the nature of the enzymatic and/or metabolic process remains to be identified, CFDA-SE reactivity can now be considered a new marker of the storage lesion. The segregation of this defect in the morphologically altered RBC strongly suggests that only a subpopulation of RBC is severely altered during storage, possibly corresponding to the older RBC present in the concentrate at the beginning of storage, as previously suggested by others (Tuo et al., 2014; Mykhailova et al., 2020).

A classic approach to study the accumulation of storage lesions is to compare groups of different storage durations (Paglia et al., 2016). Another possible approach is by using a property that evolves during storage and that allows the implementation of an enrichment strategy. Cell density is known to increase during storage (Diaz et al., 1996) and permits to study the evolution of cellular properties in each cell fraction along storage (Owusu et al., 2013; Ciana et al., 2017) or even to first separate different subpopulations and to evaluate the evolution of storage lesion markers in each fraction (Mykhailova et al., 2020). Microfluidics can also be used to enrich in RBC based on their margination capacity in circulation (Chen et al., 2018).

Imaging flow cytometry and scanning electron microscopy show that the CFDA-SE staining protocol presented here offers the possibility to obtain >95% pure subpopulations of SME (CFSE*^high^*) by flow cytometry-based sorting. Pure subpopulations of SME or morphologically normal erythrocytes would greatly facilitate the exploration of the intrinsic alterations of RBC inducing their clearance.

In our study, CFSE*^high^* erythrocytes were slightly more numerous than SME. This difference may stem from the presence of a few additional altered erythrocytes appearing upon incubation for staining, not detected when experiments are performed on RBC sampled directly from refrigerated blood bag. These erythrocytes, unable to maintain their cellular homeostasis at 37°C, would become SME and remain highly fluorescent due to their enzymatic and/or metabolic lesions. Indeed, an overnight incubation step at 37°C was shown to expose some RBC lesions induced by hypothermic storage (Burger et al., 2013).

Roussel et al. (2021) recently showed that SME accumulate during storage, reaching a mean proportion of 24% of the entire RBC population at day 42 of storage. The proportion of SME in circulation decreased rapidly when perfusing long-stored red cell concentrates in an *ex vivo* model of human spleen. Similarly, a mouse model showed decreased transfusion recovery of SME when compared to morphologically normal long-stored RBC. Furthermore, the proportion of SME in red cell concentrates negatively correlated with transfusion recovery in healthy volunteers using ^51^Chr-labeling procedure, confirming that SME quantification is a physiologically relevant marker of RBC storage quality, predictive of transfusion recovery.

Even though transfusing short-stored, as compared with standard-issue, red cell concentrates does not reduce mortality (Lelubre and Vincent, 2013; McQuilten et al., 2018, 2020), storage duration is associated with decreased transfusion recovery (Luten et al., 2008; Rapido et al., 2018). Suboptimal transfusion recovery is expected to increase the number of red blood cell concentrates needed to restore hemoglobin concentration. Along this line, transfusion-induced hemoglobin increments are significantly decreased when red cell concentrates are stored for longer durations (Hunsicker et al., 2018; Roubinian et al., 2019; Rydén et al., 2019) and may be associated with donor genetic factors (Roubinian et al., 2022). Pretransfusion control of SME, and elimination of lower-quality products, would be particularly beneficial to chronically transfused patients with sickle cell disease, thalassemia or myelodysplastic syndromes. Transfusion-related iron overload is indeed a major cause of morbidity and mortality in these patients and the need to provide more transfusions could have significant adverse consequences.

Quantification of SME using IFC is a label-free, operator-independent, quantitative, reproducible and simple method that can be achieved in a short time. Classical microscopy techniques are effective and reliable to quantify the evolution of RBC morphology but often require long experiments and analysis by qualified technicians. Automatic morphology classification approaches by machine-learning is an active field of research that can be used, for example, to analyze images acquired with a confocal microscope or IFC (Doan et al., 2020; Simionato et al., 2021). The use of IFC and/or artificial-intelligence driven analysis promises a rapid evolution of accurate and fast methods of morphological characterization in the research field. They, however, necessitate specialized equipment, which are not yet present in a majority of clinical hematology laboratories.

Quantification of CFSE*^high^* RBC is also operator-independent and reproducible. It does not directly provide information regarding RBC morphology, but accumulation of CFSE*^high^* erythrocytes was strongly correlated to the proportion of SME detected using IFC. Quantification of CFSE*^high^* RBC only requires a standard flow cytometer: a technology available in both clinical and research laboratories. The staining protocol is fairly simple and therefore has the potential to be adapted and standardized for use in the quality control of red cell concentrates.

Carboxyfluorescein Diacetate Succinimidyl Ester staining reactivity is a new marker of storage lesion that allows quantification and sorting of morphologically altered stored RBC. Future research using this method will explore the SME cellular properties and should help better understand the storage lesion and RBC senescence.

## Data Availability Statement

The raw data supporting the conclusions of this article will be made available by the authors, without undue reservation.

## Ethics Statement

Ethical review and approval was not required for the study on human participants in accordance with the local legislation and institutional requirements. Written informed consent for participation was not required for this study in accordance with the national legislation and the institutional requirements.

## Author Contributions

MM, SP, YH, SG, and MD performed the experiments. MM, SP, YH, SG, MD, CR, OH, PR, PB, and PA analyzed the data. MM, CR, OH, PB, and PA designed the research. MM, SP, and PA wrote the manuscript. PB edited the manuscript. All authors read and approved the manuscript.

## Conflict of Interest

PA is funded in part by New Health Science. MM, SP, MD, CR, PB, and PA declare that the European patent application EP21306765 was filed on December 12th, 2021. The remaining authors declare that the research was conducted in the absence of any commercial or financial relationships that could be construed as a potential conflict of interest.

## Publisher’s Note

All claims expressed in this article are solely those of the authors and do not necessarily represent those of their affiliated organizations, or those of the publisher, the editors and the reviewers. Any product that may be evaluated in this article, or claim that may be made by its manufacturer, is not guaranteed or endorsed by the publisher.

## Supplementary Material

Supplementary Figure 1 SME that accumulate during pretransfusion storage are intensely fluorescent. **(A)** Representative projected surface area frequency plot, obtained by imaging flow cytometry (IFC), of unstained (black line) and CFDA-SE stained (purple line) long-stored RBC. The bimodal distribution shows the segregation into subpopulations of either SME (orange) or normal erythrocytes (blue) subpopulations by the nadir point of the bimodal distribution. **(B)** Comparison of the mean projected surface area of SME and normal erythrocytes subpopulation determined as in **(A)**, from eight red cell concentrates (RCC) stored 42 days. **(C)** Representative CFSE intensity frequency plots of unstained (black line) and the SME (orange line) and normal erythrocytes (blue line) subpopulations previously selected in **(A)**. **(D)** Comparison of the mean CFSE fluorescence intensities of each SME and normal erythrocytes subpopulations determined as in **(C)** obtained from eight RCC stored 42 days. Results are presented as mean ± SD in **(B,D)** and tests of Wilcoxon for non-parametric and paired data were applied to compare groups between each other (^**^*p* = 0.0078).

Supplementary Figure 2 Gating strategies to quantify morphologically altered CFSE*^high^* and morphologically normal CFSE*^low^* subpopulations of stored RBC. **(A)** Representative imaging flow cytometry (IFC) dot plot of projected surface area and fluorescence intensity of CFDA-stained RBC after 42 days of storage. Selection using only CFSE fluorescence intensity (vertical dotted black line) shows that **(B)** CFSE*^low^* subpopulation contains some SME and that **(C)** CFSE*^high^* subpopulation contains morphologically normal erythrocytes. **(D)** Selection on morphological (projected surface area by IFC) and fluorescence (CFSE intensity) criteria (oblique dotted black line) improves the gating specificity.
