# Supplementary figures and images for: Storage-Induced Micro-Erythrocytes Can Be Quantified and Sorted by Flow Cytometry
Source: Front Physiol. 2022 Feb 23;13:838138. doi: 10.3389/fphys.2022.838138 (PMC8906515; doi:10.3389/fphys.2022.838138)

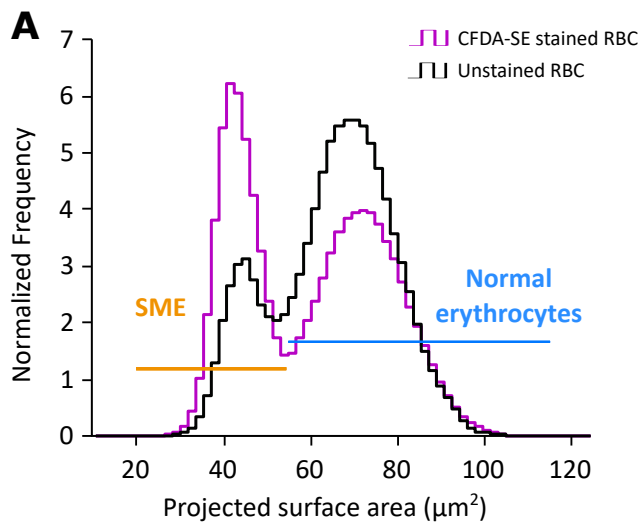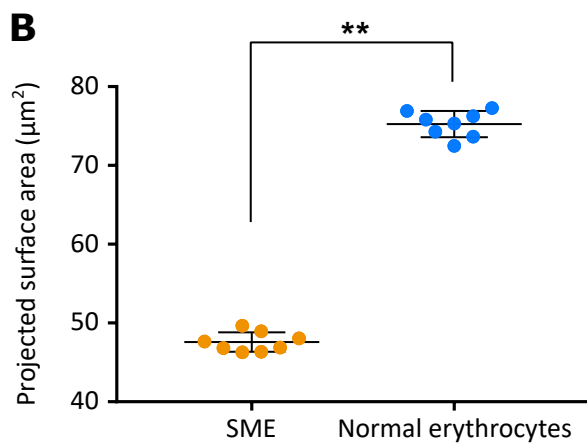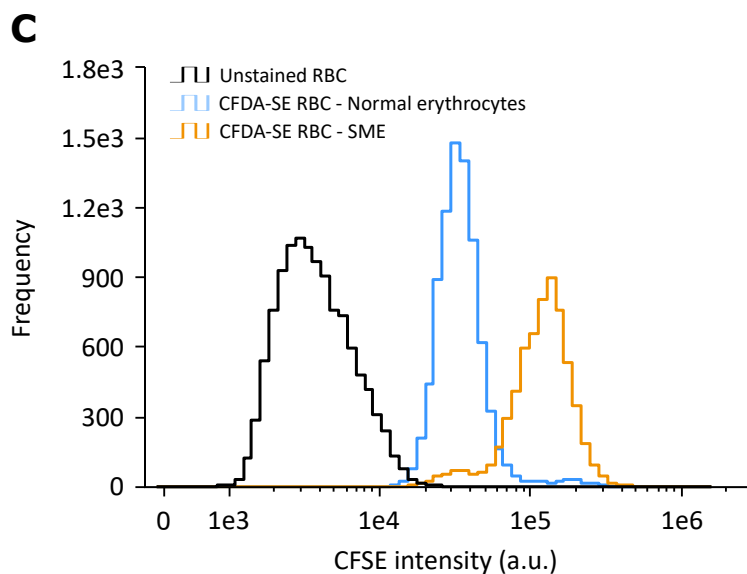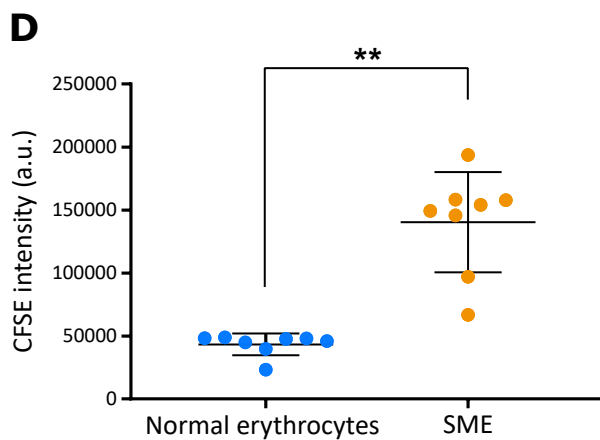

Supplement: Supplementary Figure 1 — SME that accumulate during pretransfusion storage are intensely fluorescent. (A) Representative projected surface area frequency plot, obtained by imaging flow cytometry (IFC), of unstained (black line) and CFDA-SE stained (purple line) long-stored RBC. The bimodal distribution shows the segregation into subpopulations of either SME (orange) or normal erythrocytes (blue) subpopulations by the nadir point of the bimodal distribution. (B) Comparison of the mean projected surface area of SME and normal erythrocytes subpopulation determined as in (A), from eight red cell concentrates (RCC) stored 42 days. (C) Representative CFSE intensity frequency plots of unstained (black line) and the SME (orange line) and normal erythrocytes (blue line) subpopulations previously selected in (A). (D) Comparison of the mean CFSE fluorescence intensities of each SME and normal erythrocytes subpopulations determined as in (C) obtained from eight RCC stored 42 days. Results are presented as mean ± SD in (B,D) and tests of Wilcoxon for non-parametric and paired data were applied to compare groups between each other (**p = 0.0078). [file Image_1.pdf]

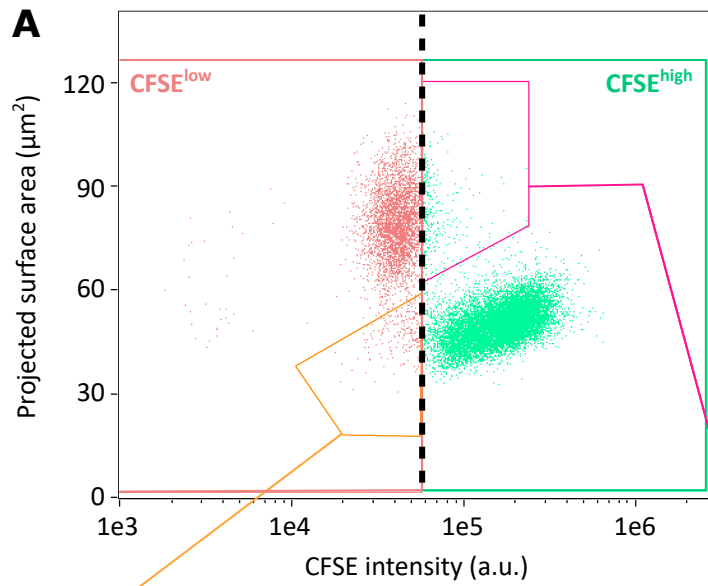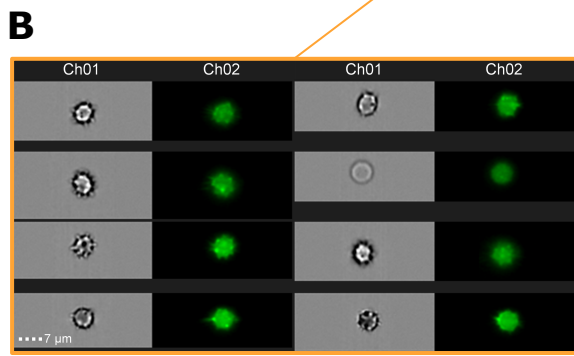

Contaminants CFSE<sup>low</sup> low area

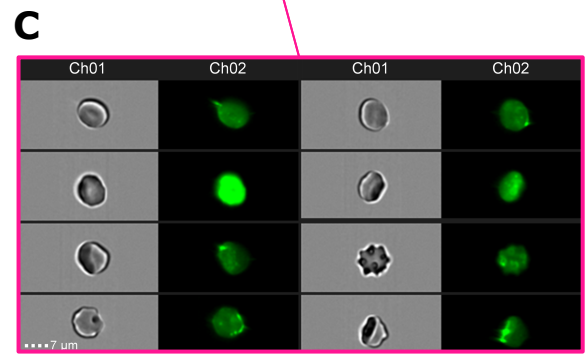

Contaminants CFSE<sup>high</sup> high area

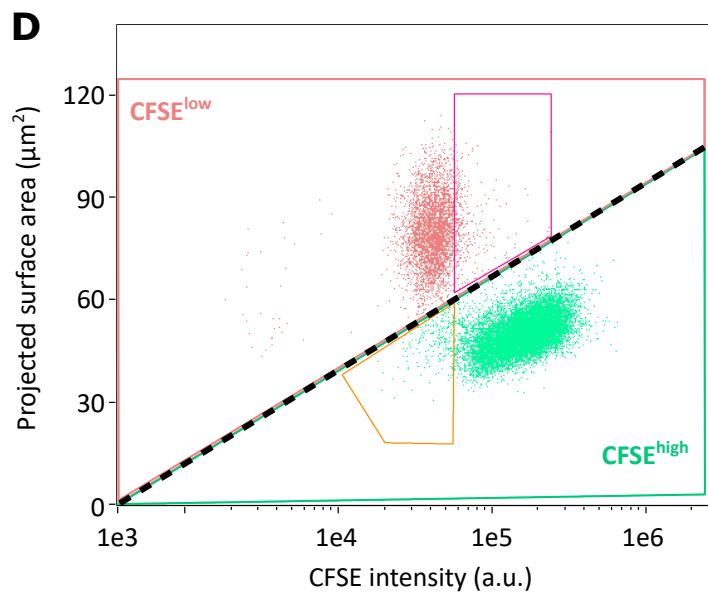

Supplement: Supplementary Figure 2 — Gating strategies to quantify morphologically altered CFSEhigh and morphologically normal CFSElow subpopulations of stored RBC. (A) Representative imaging flow cytometry (IFC) dot plot of projected surface area and fluorescence intensity of CFDA-stained RBC after 42 days of storage. Selection using only CFSE fluorescence intensity (vertical dotted black line) shows that (B) CFSElow subpopulation contains some SME and that (C) CFSEhigh subpopulation contains morphologically normal erythrocytes. (D) Selection on morphological (projected surface area by IFC) and fluorescence (CFSE intensity) criteria (oblique dotted black line) improves the gating specificity. [file Image_2.pdf]
